# Comparison of different quantification methods for ^18^F-fluorodeoxyglucose-positron emission tomography studies in rat brains

**DOI:** 10.6061/clinics/2019/e1273

**Published:** 2019-09-19

**Authors:** Silvana Prando, Camila de Godoi Carneiro, Cecil Chow Robilotta, Marcelo Tatit Sapienza

**Affiliations:** I Centro de Medicina Nuclear, Faculdade de Medicina FMUSP, Universidade de Sao Paulo, Sao Paulo, SP, BR; II Instituto de Fisica, Universidade de Sao Paulo, Sao Paulo, SP, BR

**Keywords:** Quantification, [18F]FDG, SUV, FUR, K_i_

## Abstract

**OBJECTIVES:**

This study aimed to evaluate several methods to estimate glucose consumption in the male Wister rat brain as measured by PET.

**METHODS:**

Fourteen male Wistar normoglycemic rats were studied. The input function consisted of seventeen blood samples drawn manually from the femoral artery. Glucose uptake values were calculated using the input function resulting from the arterial blood samples and the tissue time-activity curve derived from the PET images. The estimated glucose consumption rate (K_i_) based on the 2-tissue compartment model (2TCM) served as the standard for comparisons with the values calculated by the Patlak analysis and with the fractional uptake rate (FUR), standardized uptake value (SUV) and glucose corrected SUV (SUV_glu_).

**RESULTS:**

No significant difference between the standard K_i_ and the Patlak K_i_ was observed. The standard K_i_ was also found to have strong correlations and concordance with the K_i_ value estimated by the Patlak analysis. The FUR method presented an excellent correlation with the K_i_ value obtained by the 2TCM/Patlak analyses, in contrast to the SUV or SUV_glu_.

**CONCLUSIONS:**

From a methodological point of view, the present findings confirm the theoretical limitations of the cerebral SUV and SUV_glu_ as a substitute for K_i_ in the estimation of glucose consumption in the brain. Our data suggest that the FUR is the surrogate to K_i_.

## INTRODUCTION

Positron emission tomography (PET) combined with 18F-fluorodeoxyglucose ([18F]FDG) is a powerful tool for investigating brain metabolism *in vivo* 1-4. PET is a medical imaging technique that is based on the administration of labeled drugs with positron-emitting radioisotopes, where the chemical form of the radiopharmaceutical is designed to provide information on tissue biochemistry rather than anatomy. The images are formed by the detection of two opposing gamma rays that are produced in the annihilation process between the positron and the electron. Thus, PET provides a means to measure the local concentrations of positron emitters and to reconstruct the images of the radiopharmaceutical distribution in the brain. The images obtained from the brains of small animals through PET are often evaluated using several quantitative analysis approaches.

The gold standard in PET quantification is the compartment model. Well-established compartmental models in PET include those used for quantification of blood flow 5, the cerebral metabolic rate for glucose 6,7 and neuroreceptor binding 8.

However, these particular models require the acquisition of dynamic images and an arterial or plasma blood input function, with the number of tissue compartments dictated by the physiological, biochemical and physiological parameters that are properties of the system being studied.

A simplification of the compartmental methods is the Patlak graphical analysis 9,10. The main advantage of this approach is the possibility of acquiring PET images at a late stage, where the system is in the steady state, after the injection of the radiopharmaceutical. The Patlak standard linear graphical analysis is a robust modeling approach and allows a direct estimation, from the reconstructed PET images and the input function, of the influence of the tracer K_i_ and blood volume V 9.

However, the application of compartmental or Patlak models is technically challenging, somewhat complicated and not sufficiently practical for routine use, even in small animals 11. Simplified quantification approaches have been introduced to overcome these challenges.

The standardized uptake value (SUV) is the parameter frequently used to measure [18F]FDG uptake and to distinguish between areas with altered metabolism concerning the normal brain 12,13. Its use is related to ease of implantation, without the need to acquire dynamic images or obtain the concentration of the radiopharmaceutical in the blood (the function of entrance). However, the SUV is strongly dependent on the time of injection and the current metabolic state of each animal. Its outcome can also be affected by factors including animal management and biological or technical considerations 13-17. Anesthesia is a factor that is present in the vast majority of preclinical studies, and it affects glucose metabolism, frequently causing hyperglycemia 18-20. In hyperglycemic animals, Woo et al. 21 suggest that the SUV can be corrected through a normalized serum baseline glycemia value for the population mean.

An alternative to SUV correction for basal glycemia is the use of the fractional uptake rate (FUR), which normalizes the plasma variability of the population through the acquisition of the concentration of the radiopharmaceutical in the blood between the time of injection and the end of the image acquisition 22,23. The FUR is considered an approximation of the slope (K_i_) obtained by the Patlak linearization technique for a delay time after the injection 24.

In this study, we used the [18F]FDG radiopharmaceutical to evaluate the methods of quantification of glucose metabolism measured by PET in small animals. Net glucose consumption values (K_i_) were obtained through the compartmental (2TCM) and Patlak method and were compared with the values obtained by the SUV, SUV_glu_, and FUR methods.

## MATERIALS AND METHODS

### Animals

The procedures described in the present work are part of a small-animal PET experiment examining the effects of anesthetics on cerebral glucose metabolism 25. The current work addresses the issue of comparing different quantification methods. Only the animals with 17 samples of arterial blood were analyzed. We retrospectively analyzed fourteen male Wistar rats obtained from the Animal Facility Center of the Medical School at the University of São Paulo, Brazil. The animals were given at least 7 days to acclimate to the vivarium of the Laboratory of Medical Investigation - LIM 43 in a climate-controlled room with a 12-hour circadian cycle and free access to food and water. On the day of the experiment, the animals were subjected to surgery for implantation of a cannula into the femoral artery and to PET under anesthesia with isoflurane or ketamine-xylazine.

All procedures were in accordance with ethical principles adopted by the Brazilian College of Animal Experimentation and approved by the Ethical Committee for Animal Research of School of Medicine, University of São Paulo (protocol 026/14).

### Anesthesia

Prior to the PET scan, a mixture of 5% isoflurane (FORANE, Baxter Healthcare Corporation) and medical air was used to anesthetize the animals, which were maintained under anesthesia at 1.5 – 2.0% isoflurane (n=5) or with an intraperitoneal injection of ketamine (n=9) (100 mg/kg) associated with xylazine alpha2-agonist (10 mg/kg) (DOPALEN, Sespo Indústria e Comércio Ltda; ANASEDAN, Sespo Indústria e Comércio Ltda).

Animals anesthetized with intraperitoneal ketamine injection associated with alpha2-xylazine agonist received a standardized dose by weight during induction and a second fractionated dose every 30 minutes. In total, each animal received two doses of the anesthetic.

A PE50 cannula (0.58 mm internal diameter and 0.96 mm outside diameter, Becton Dickinson) was inserted into the femoral artery to obtain arterial blood samples 26.

The mean time between the onset of induction and [18F]FDG injection was 30 minutes. The total experiment time was 90 minutes. The animals remained on a surface heated to 37°C for the experimental duration to avoid hypothermia. During induction, the baseline serum weights and glycemia levels of the animals were measured.

### Positron emission tomography

After the general procedures, image acquisition and arterial blood collection were started synchronically with the injection of the radiopharmaceutical. A bolus injection of 48.9±3.5 MBq of [18F]FDG was administered manually into the penile vein.

The dynamic images of one bed position were acquired in list mode with a LabPET 4 system (Gamma Medica-Ideas, Northridge, CA) 27 over 1 hour. The acquired data were divided into 20 images (six series with the following numbers and durations: 8/30 s, 2/60 s, 2/120 s, 2/150 s, 3/300 s, and 3/600 s). Reconstruction was performed using the OSEM-2D method (20 iterations, 4 subsets, FOV=60 mm, high resolution mode), which resulted in 0.500 x 0.500 x 0.597 mm voxels. The data were corrected for radionuclide decay and random coincidences.

Simultaneous with PET image acquisition, 17 arterial blood samples were collected from the femoral artery at the PET acquisition timepoints 0, 5, 10, 20, 30, 40, 50, 60, 90, 120, 180, 300, 450, 600, 900, 1800 and 3600 s. The count rate per minute for an aliquot of 50 µL of sampled blood was measured with a gamma scintillation counter (Wallac Wizard 3, Perkin-Elmer), and the counts obtained were corrected for radionuclide decay and converted to activity concentration (kBq/mL) based on the gamma scintillation counter calibration factor, which was previously determined on the day of the experiment. The activity levels of the samples were corrected for the accumulated [18F]FDG activity in red blood cells following the method formulated by Wu et al. 28. The time-activity curve (TAC) for blood was corrected to a delay between injections and the start of acquisition in PMOD software, version 3.4 (PMOD Technologies Ltd., Zurich, Switzerland). The result was used as an arterial input function.

### Data analysis

#### Standardized uptake value

The set of dynamic images for each animal was automatically coregistered using the T2-MRI template available in PMOD software, version 3.4 (PMOD Technologies Ltd., Zurich, Switzerland), and the mean SUV was generated by projection volumes of interest (VOIs) on the dynamic images.

The mean SUV 29 was calculated using the following equation:



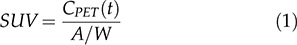


where C_PET_(t) is the mean [18F]FDG concentration in dynamic images; A is the [18F]FDG activity injected into the animal corrected for radionuclide decay and residual remaining in the syringe; and W is the animal's body weight.

The whole brain, consisting of the thalamus, cerebellum, hypothalamus, hippocampus, caudate nucleus, putamen, and cortex as defined in Schiffer's brain atlas 30 available in the PMOD software, was designated the VOI, and the VOI was used to obtain the mean SUV.

To calculate the metabolism value, we considered the last 30 minutes of the study.

The SUV_glu_ was generated by multiplying the SUV by the basal serum glycemia level of each animal.

#### Fractional uptake ratio

The FUR was obtained according to equation 2 and represents the ratio of tissue activity at time t and the integral of plasma activity from time 0 to t 22-24:



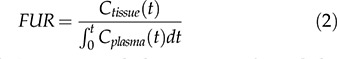



For the FUR calculation, we used the concentration of the activity per voxel in 45 minutes, which corresponds to the mean time of the last 30 minutes of the study, and the input function from 0 to 45 minutes.

#### Kinetic analysis

Kinetic analysis was performed in PMOD software, version 3.4 (PMOD Technologies Ltd., Zurich, Switzerland) using the compartmental method of irreversible tissues (2TCM) 6 and Patlak 9,10.

Whole-brain TACs were obtained by applying the VOI described above.

#### Two-tissue compartment model (2TCM)

**Figure 1:**
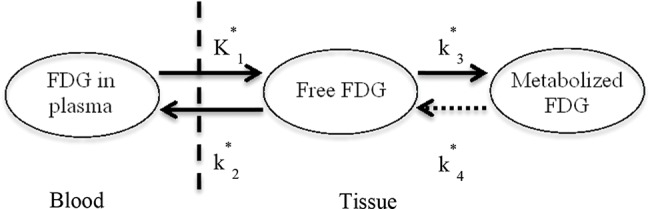
Two-tissue compartment model describing [18F]FDG pharmacokinetics, including the blood-to-tissue [18F]FDG transport rate (K_1_), tissue-to-blood [18F]FDG transport rate (k_2_), phosphorylation to [18F]FDG-6-phosphate rate (k_3_), and dephosphorylation rate (k_4_).

In the 2TCM 6 (Figure 1), it is assumed that the [18F]FDG is exchanged between the compartments, where each compartment represents a homogeneous, physiological or biochemical entity, and the rates at which the [18F]FDG is transferred between the compartments are described by first-order differential equations, namely, equations 3 and 4. The 2TCM requires dynamic imaging from the time of injection and, in general, a TAC to measure the concentration of the radiopharmaceutical in the plasma as a function of the time of the study. The advantages of this method are its reliability and its independence of examination time or plasma clearance, in contrast to the SUV. Because 2TCM can estimate kinetic parameters, we can determine glucose transport and hexokinase activity for each region of interest in the image.



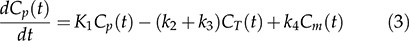




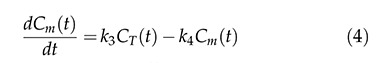


The difficulty in solving the differential equations above is that the values for the transfer rates for glucose (K1, k2, and k3) must be determined. Direct measurement of transfer rates to glucose is very complicated, so we used [18F]FDG-PET measurements to estimate these values from [18F]FDG.</p>

Initially, the cerebral metabolic rate of glucose cMRglu is expressed in terms of [18F]FDG transfer constants (*K*1*, *k*2*, *k*3* and *k*4*) and can be expressed in the form of equation 5:



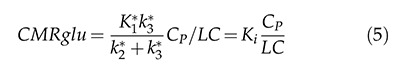


The cMRglu equation is a simple equation involving the transfer rates of [18F]FDG (*K*_1_*, *k*_2_* and *k*_3_*), the blood glucose concentration C_P_ during the equilibrium phase and the lumped constant, representing the difference between the metabolism of glucose and [18F]FDG. The LC value depends on the basal serum glucose level of the animal 31 and the region of the brain 32.

Since we used different anesthetics in this work and did not determine the appropriate lumped constant for each anesthetic protocol, we chose to use *K_i_* as the metabolic rate for the whole brain.

The graphing methods allow appropriate estimations of certain combinations of micro parameters by transforming the estimation equations on which the compartmental models are based. There are two types of graphing methods, namely, the Patlak method and the Logan method, which can be applied to irreversible or reversible substances, respectively.

In the Patlak linearization method, one should presume that at least one compartment contains the radiopharmaceutical irreversibly and that the examination time is sufficient for the plasma and tissue compartments to reach steady state. In the case of [18F]FDG, the radiopharmaceutical is transformed into [18F]FDG-6-PO_4_ and remains irreversibly retained within the neurons. In these circumstances, only the accumulation of the tracer in irreversible compartments affects the apparent volume of distribution.

After a certain time t *, which depends on the plotter, the subject and the ROI, the relationship of the TAC of the tissue C_T_ (t) and C_T_ of the plasma C_P_ (t) (y-axis) with the ratio of the integral to the instantaneous value of C_P_ (t) (x-axis) becomes linear, as expressed by equation 6:



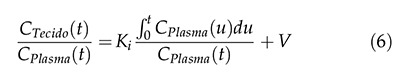


where K_i_, in equation 7, indicates the rate at which the tracer is irreversibly retained and can be calculated from the above equation using a simple linear estimation procedure.



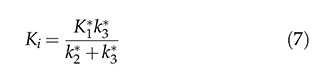


This method requires the acquisition of dynamic images beginning 15 to 30 minutes after the tracer injection and arterial blood samples. Due to the linearity of the above equation, the method is much faster and less sensitive to noise than the 2TCM and is therefore suitable for voxel applications. For [18F]FDG, we can calculate the cerebral metabolic rate of glucose cMRglu (μmol/min/100 g) from K_i_ by equation 8:



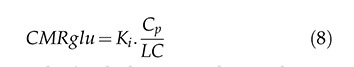


For each animal, we obtained the rate of net glucose uptake (K_i_).

#### Statistical analysis

The results of the study were analyzed by means of the SPSS 22.0 statistical package (SPSS Inc., Chicago, IL, USA). The Shapiro-Wilk test was used to verify the normality of variables.

Pearson's correlation coefficient was used to analyze the correlation between all the methods used in this study and classified as weak (0.05<*p*≤0.01), strong (0.01<*p*≤0.05) or strongest (*p*<0.01).

The Bland-Altman method 33 and the Lin correlation coefficient 34 were used to analyze the agreement between K_i_ estimated by the 2TCM and Patlak models and the SUV and SUV_glu_ models.

## RESULTS

All variables used in this study showed a normal distribution according to the Shapiro-Wilk test. Table 1 shows the mean, standard deviation and confidence interval for the baseline serum glycemia, weight and injected activity of the total sample studied.

**Figure 2:**
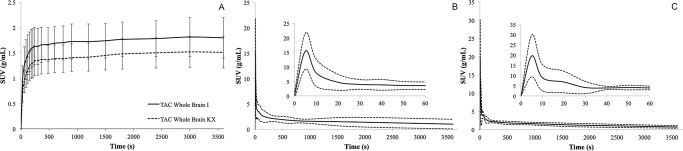
Representative time-activity curves of brain and plasma input from the ketamine/xylazine or isoflurane group. A) Average [18F]FDG time-activity curves of the brain. Data are expressed as the standardized uptake value (mean ±SD). B) [18F]FDG time-activity curves for whole blood from the ketamine/xylazine group. The dashed line represents the confidence interval, and the solid line represents the mean. C) [18F]FDG time-activity curves for whole blood from the Isoflurane group. The dashed line represents the confidence interval, and the solid line represents the mean.

Figure 2 shows the TACs for the whole brain (a) and input functions for animals anesthetized with ketamine/xylazine (b) or isoflurane (c).

**Figure 3:**
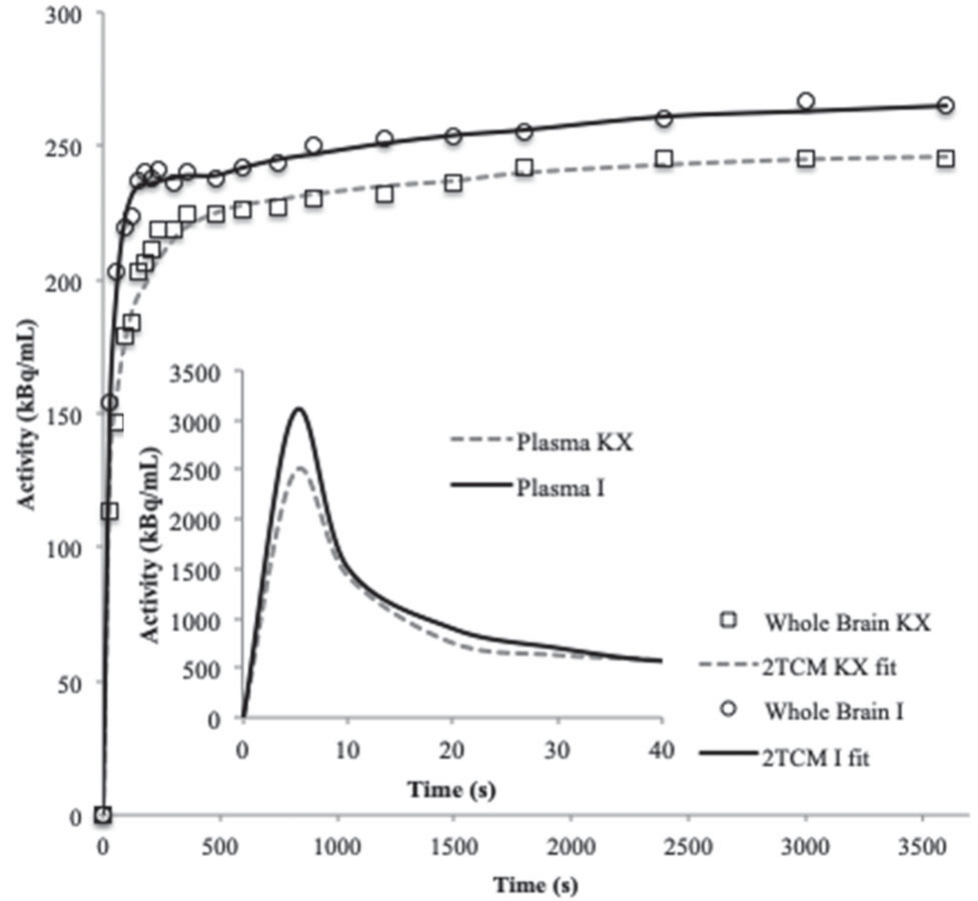
Representative (n=1) time-activity curve and 2TCM fit for the whole brain and time-activity curve of the whole blood of an animal from the ketamine/xylazine or isoflurane group.

Figure 3 shows a representative TAC and 2TCM fit for the whole brain and the input function for each anesthetic. The visual inspection revealed a satisfactory fit between the data and the 2TCM.

Their mean and respective error values are shown in Table 2.

**Figure 4:**
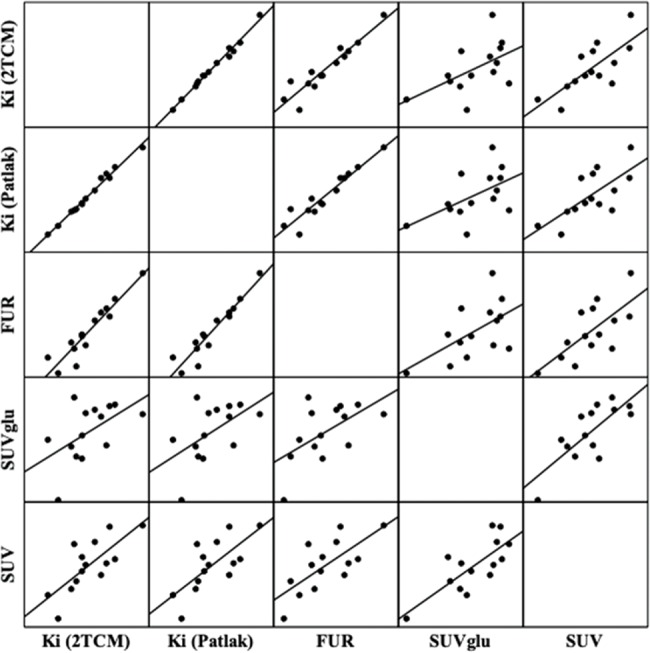
Correlation graphs and linear regression matrix between the methods of quantification of the cerebral metabolism of glucose measured by PET-[18F]FDG. The solid lines represent the regression lines. Ki: net glucose consumption; 2TCM: two-tissue compartment model; FUR: fractional uptake value; SUVglu: standardized uptake value corrected by glucose, SUV: standardized uptake value.

The correlations between the methods used can be visualized in Figure 4 and in Table 3. There were significant positive correlations between K_i_ (Patlak) and K_i_ (2TCM) (r=0.9935, *p*<0.001); FUR and K_i_ (Patlak) (r=0.9472, *p*<0.001); FUR and K_i_ (2TCM) (r=0.9385, *p*<0.001); SUV_glu_ and SUV (r=0.7671, *p*=0.001); SUV and K_i_ (2TCM) (r=0.7336, *p*=0.002); SUV and K_i_ (Patlak) (r=0.7123, *p*=0.004); SUV and FUR (r=0.7095, *p*=0.004); SUV_glu_ and FUR (r=0.5752, *p*=0.031); and SUV_glu_ and K_i_ (Patlak) (r=0.045, *p*=0.545) (r=0.045, *p*=0.545).

**Figure 5:**
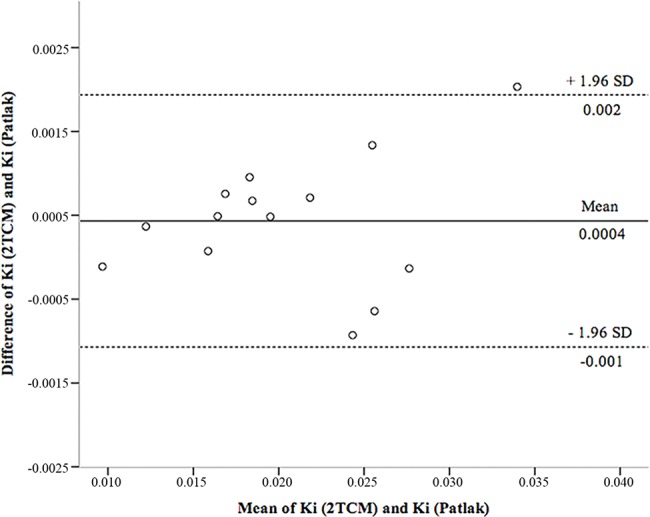
Representative Bland-Altman plot of the agreement in the K_i_ estimation between the Patlak and reference values obtained from the 2TCM. The dashed line represents the confidence interval, and the solid line represents the bias. Ki: net glucose consumption; 2TCM: two-tissue compartment model; SD: standard deviation.

Figure 5 shows the Bland-Altman scatter plot used to determine the agreement between K_i_ obtained by the 2TCM and Patlak analyses. It was observed that the methods were concordant and with reduced cases of dispersion beyond the upper and lower limits of the standard deviations of the means.

In Table 4, Lin's correlation coefficients indicate that there is excellent agreement between K_i_ (2TCM) and K_i_ (Patlak).

## DISCUSSION

In this article, we chose not to use the cerebral metabolic rate of glucose consumption (cMRglu) in the comparison of the methods. Methods using a tracer for the measurement of cMRglu require knowledge of the value of the lumped constant, which explains the difference in enzymatic kinetics of transport and phosphorylation between tracer and glucose. Although the relative activity of an enzyme for the tracer and natural substrate is generally constant and predictable, it has been reported previously that changes in basal glycemia levels alter the value of the lumped constant 31,35,36. As the anesthetics used in this study alter glycemia levels, we chose not to use cMRglu because we believed that we would be introducing a source of error that would impair the comparison of the methods.

In the absence of cMRglu, we chose to use K_i_ as the reference, which represents the glucose uptake rate and incorporates both internal net transport and tracer trapping in the tissue. K_i_ can be calculated by fitting the compartment model; in this case, 2TCM or the graphical approach of Patlak can be used to measure K_i_ without making assumptions about the metabolic compartments 9.

The compartmental model, namely, the 2TCM, is considered the gold standard in quantification because it contains a larger amount of acquired data and is subject to fewer assumptions. However, our results demonstrate that the 2TCM and Patlak graphical approach are strongly correlated (r=0.9935, *p*<0.001) and are concordant (CC=0.991) with each other. This result indicates that the K_i_ obtained by Patlak can be used in the quantification of the cerebral metabolism of small animals in place of the 2TCM. The primary advantage of adopting the Patlak method in the experimental routine lies in the fact that the method is less sensitive to the noise of the initial part of the study and requires a simpler acquisition protocol that may eventually be translated into clinical practice 37.

As an alternative to compartmental methods, simplified methods have been proposed over the years to facilitate quantification by avoiding dynamic acquisitions and arterial blood collection. The best-known simplified method today is the standardized uptake value, namely, the SUV.

The SUV is an estimate of the glucose consumption rate (K_i_) related to the concentration of radiopharmaceutical activity in a voxel, acquired by the equipment, and the concentration of activity in the animal. The accuracy of this estimate depends on several technical, physiological and methodological factors 16,37,38.

The acquisition and processing parameters affect the estimates of metabolism and must be standardized to ensure that the image reflects the behavior of the tracer, producing an accurate and reproducible result.

Factors including hyperglycemia, competition and metabolic differences between glucose and [18F]FDG affect all methods and should not be seen as a technical/methodological disadvantage because they are intrinsic to the animal. Although compartmental methods attempt to correct transport and metabolism variations through the lumped constant, to date, it has not been possible to eliminate the associated uncertainties 32,39-41. One of the ways found to approach the SUV of the cMRglu was to correct it by means of the basal serum glycemia level, thus generated the SUV_glu_ 21. Our data show that the SUV_glu_ has no advantage over the SUV, FUR or K_i_, as seen in the correlation coefficient between them (r=0.7671; r=0.5752; r=0.5456). The correlation between the SUV_glu_ and K_i_ decreased with the coefficient obtained between the SUV and K_i_ (0.5456 *vs.* 0.7336). A plausible and already verified explanation with patients is related to the method of measurement of the basal serum glucose concentration. When methods known as bedside methods are used, as in this article, the reproducibility of the blood glucose measurement is 10 to 15%, in contrast to that of the hexokinase method, which is a more accurate measurement method. The use of these methods can lead to SUV variations of up to 30% 38. Therefore, SUV correction is not recommended unless an accurate method of measuring blood glucose is available 42.

In general, the main parameters related to glucose uptake and that may alter the result are the rate of glucose extraction by the organ of interest and other organs, perfusion, injected activity and volume of distribution 15,17. The main limitations of the SUV related to errors including extravasation, recording of the injected activity, the variation of the absorption due to other organs and differences between plasma and body volume are corrected in the K_i_ calculation by the inclusion of the input function.

In the images obtained by PET, the region of interest used for quantification contains information on the concentration of metabolized and nonmetabolized [18F]FDG present in the vascular and extravascular compartments. In addition to accounting for the amount of unmetabolized [18F]FDG, the SUV takes into account that metabolism is related to the body weight, lean mass or body surface area of the animal. The total weight is the most common normalization method for the calculation of the SUV. However, there are differences between the plasma and body volume ratio in laboratory animals. Obese, dehydrated animals that have lost a large amount of body mass or that are very hydrated exhibit an altered relationship between the plasma and body volumes that can influence the outcome of the SUV. If the normalization of the dose injected by weight is not proportional to the integral of the [18F]FDG concentration in the plasma, namely, the dose of bioavailable [18F]FDG provided by the input function during the study time, the accuracy will be impaired. Additionally, one should keep in mind that quantitatively measuring uptake is not the same as measuring a pathophysiological process quantitatively.

It is known that hyperglycemia decreases [18F]FDG arterial inflow due to faster blood clearance and that the same injected dose of [18F]FDG does not guarantee the same arterial inflow of [18F]FDG 22. To minimize this problem, one can calculate the level of glucose consumption through the FUR that represents normalized glucose uptake by [18F]FDG arterial inflow and may neglect the influence of uptake by other [18F]FDG organs and of the patient's body weight 23-25. Our results, shown in Table 3 and Figure 4, indicate that there is an excellent correlation between the FUR and K_i_ (Patlak) (r=0.9472, *p*<0.001) and FUR and K_i_ (2TCM) (r=0.9385, *p*<0.001). The FUR has a better correlation with the more complex models than the SUV, which allows us to affirm that the method is an adequate substitute for the K_i_ when the objective is to make comparisons between normal and pathological states or between animals. The major advantage of the FUR method over the 2TCM and Patlak analysis is the use of static images for quantification, which would make translation easier for clinical studies.

However, if the aim of the study is primarily to identify relative metabolic heterogeneities, the SUV can be used as long as the acquisition and processing protocol is rigidly established.

From the methodological point of view, the present findings confirm the theoretical limitations of the SUV and cerebral SUV_glu_ as a surrogate for K_i_ in the estimation of glucose consumption in the brain. Our data suggest that the FUR is the surrogate for K_i_ when dynamic acquisition is not possible.

## AUTHOR CONTRIBUTIONS

Prando S was responsible for the study concept and design. Prando S and Carneiro C contributed to the acquisition of the animal data. Prando S, Sapienza MT and Robilotta CC assisted with the data analysis and interpretation of the findings, drafted the manuscript and critically revised the manuscript for important intellectual content.

## Figures and Tables

**Table 1:** Mean, standard deviation and confidence interval of serum basal glycemia (mmol), weight (g) and injected activity (MBq).

	Mean	SD	95% CI
Glycemia (mmol)	7.11	1.07	6.59-7.78
Weight (g)	343.67	55.55	312.90-374.43
Injected activity (MBq)	49.15	3.41	47.26-51.04

SD = Standard Deviation, IC = Confidence Interval.

**Table 2:** Mean, standard error mean and confidence interval of K_i_ (2TCM) (mL*(min*mL)^-1^), K_i_ (Patlak) (mL*(min*mL)^-1^), SUV, SUV_glu_ (mmol) and FUR (min^-1^).

	Mean	SEM	95% CI
K_i_ (2TCM)	0.0207	0.0018	0.0169-0.0245
K_i_ (Patlak)	0.0202	0.0017	0.0165-0.0239
FUR	0.0011	0.0001	0.0009-0.0013
SUV_glu_	2.03	0.12	1.76-2.29
SUV	1.57	0.09	1.38-1.76

SEM = Standard Error of the Mean, IC = Confidence Interval.

**Table 3:** Pearson correlation coefficient.

	K_i_ (2TCM)	K_i_ (Patlak)	FUR	SUV_glu_	SUV
Ki (2TCM)	1.0000				
K_i_ (Patlak)	0.9935	1.0000			
	*p*<0.001				
FUR	0.9385	0.9472	1.0000		
	*p*<0.001	*p*<0.001			
SUV_glu_	0.5419	0.5456	0.5752	1.0000	
	*p*=0.045	*p*=0.044	*p*=0.031		
SUV	0.7336	0.7123	0.7095	0.7671	1.0000
	*p*=0.002	*p*=0.004	*p*=0.004	*p*=0.001	

SUV: standardized uptake value; FUR: fractional uptake value; K_i_: net glucose consumption; 2TCM: two-tissue compartment model.

**Table 4:** Results of the Lin (CC) correlation coefficient of the Bland-Altman analysis (mean differences and confidence intervals).

	CC	Mean Differences (SD)	95% CI
K_i_ (2TCM) *vs.* K_i_ (Patlak)	0.991	0.000 (0.001)	(-0.0019) - 0.002

CC = Correlation Coefficient; SD = Standard Deviation; CI = Confidence Interval.
